# Laser hair removal to antiretrovirals: findings from a person‐centred care model for transgender people in India

**DOI:** 10.1002/jia2.70041

**Published:** 2025-10-08

**Authors:** Simran Shaikh, Parthasarathy Mugundu Ramien, Jade Bell, Kanchan Pawar, Allison M. McFall, Saya Okram, Ajay Enugu, Lakshmi Ganapathi, Maria Salvat Ballester, Viswanathan Arumugam, Rose Pollard Kaptchuk, Aditya Singh, Shantanu Kumar Purohit, Alex Keuroghlian, Kevin Ard, Shruti H. Mehta, Sukhvinder Kaur, Kenneth H. Mayer, Sunil Suhas Solomon

**Affiliations:** ^1^ The Johns Hopkins University School of Medicine New Delhi India; ^2^ The Johns Hopkins University School of Medicine Baltimore Maryland USA; ^3^ The Johns Hopkins Bloomberg School of Public Health Baltimore Maryland USA; ^4^ Y.R. Gaitonde Centre for AIDS Research and Education Chennai India; ^5^ Massachusetts General Hospital Boston Massachusetts USA; ^6^ Harvard Medical School Boston Massachusetts USA; ^7^ National AIDS Control Organization New Delhi India; ^8^ The Fenway Institute Boston Massachusetts USA; ^9^ USAID New Delhi India; ^10^ Beth Israel Deaconess Medical Center Boston Massachusetts USA

**Keywords:** comprehensive services, gender‐affirming care, HIV, low‐ and middle‐income country, person‐centred, transgender people

## Abstract

**Introduction:**

Transgender women (TGW) in India continue to bear disproportionate HIV burden and face persistent social, legal and structural barriers to receive gender‐affirming care.

**Methods:**

Since 2021, we established three “*Mitr*” (meaning: friend) clinics in Hyderabad, Pune and Thane, India, for transgender people with staffing primarily from the community. *Mitr* clinics provide free HIV testing and pre‐exposure prophylaxis (PrEP) on site with linkage to government antiretroviral therapy (ART) centres. They also provide free consultation for gender‐affirming hormone therapy (GAHT), subsidized laser hair removal and legal assistance. Client service utilization data were analysed using summary statistics to evaluate uptake of HIV and gender‐affirming services; correlates of HIV testing were examined using logistic regression. Semi‐structured interviews conducted at one site were used to understand barriers/facilitators of HIV testing.

**Results:**

A total of 5223 unique clients registered between March 2021 and September 2024; median age was 26 years. Most (86%) self‐identified as TGW, and 35% reported transactional sex. Most clients (70%) had not previously accessed public sector HIV services. The majority (75%) accessed *Mitr* clinics for gender‐affirming care, including laser hair removal (53%), GAHT consultations (34%) and surgical referral (26%). Over half (62%) of clients eligible for HIV testing underwent screening, of whom 6% were newly diagnosed. Accessing *Mitr* clinics for gender‐affirming surgical services was significantly associated with HIV testing receipt (aOR: 1.51; 95% CI: 1.02, 2.25). Services provided by staff from the community were a prominent facilitator for HIV testing, while stigma and disclosure concerns were notable barriers. Among 585 clients interested in and eligible for PrEP, 576 (98%) initiated PrEP, and 378 (66%) were PrEP persistent at 3 months. Of 454 clients with HIV (newly diagnosed or previously known), 392 (86%) initiated ART. As of 30 September 2024, 233 (59%) were still receiving *Mitr* clinic services and retained in HIV care; viral suppression was 98% among the 156 clients with data.

**Conclusions:**

The *Mitr* model highlights the importance of aligning programme and community priorities. The provision of gender‐affirming care attracted many clients who might not otherwise have accessed HIV services; indeed, laser hair removal served as the key entry point to HIV testing, PrEP and ART.

## 1 INTRODUCTION

Globally, transgender people experience significant disparities in access to health services, shaped by structural and psychosocial factors including stigma, discrimination, violence and legal barriers [1, 2, 3]. These inequities extend to receipt of HIV services [4, 5, 6, 7], with global estimates indicating a high burden of HIV among transgender people [8, 9, 10] and unequal outcomes across the HIV care continuum compared to the general population [11, 12, 13, 14].

Transgender communities in India [15, 16] are highly diverse and include organized groups such as Hijra communities that have evolved social structures built on systems of kinship [17]. However, laws criminalizing gender and sexual minority individuals [18] have perpetuated intersectional stigma [19, 20, 21, 22] and contributed to the marginalization of transgender people. Consistent with global findings [23, 24, 25, 26, 27, 28], transgender people in India experience minority stress and syndemics that influence health outcomes [29, 30, 31, 32, 33, 34, 35, 36, 37, 38]. Hijra/transgender women (TGW) in India have a HIV prevalence of 3.8% [39], nearly 19 times higher than the general population.

Following long‐standing advocacy, the Transgender Persons (Protection of Rights) Act was passed in 2019, recognizing equal rights of transgender people to education, employment and healthcare [40]. The Act prohibits discrimination in healthcare services, and mandates that medical facilities include gender‐affirming services to support a person's process of living and being perceived in alignment with their gender identity [40]. Despite this act, the implementation of gender‐affirming care models is rare. The delivery of public sector health services to transgender people has largely focused on HIV prevention and treatment without the integration of gender‐affirming care; prevention services are primarily delivered through the Targeted Intervention (TI) programme, while antiretroviral therapy (ART) is provided at over 700 government ART centres. HIV pre‐exposure prophylaxis (PrEP) is not available in the public sector free of charge. While medical and surgical gender‐affirming services are available in the private sector, cost limits accessibility [41, 42, 43]. Further, state‐level variability and bureaucracy limit accessibility to social welfare schemes [41, 44, 45, 46].

Multiple service delivery models globally for transgender people have paved the way for the provision of person‐centred care [47, 48, 49, 50, 51, 52, 53] (i.e. a care approach that prioritizes the values, goals and preferences of transgender people over a particular disease/diseases outcome(s)). These models are built on the principle that provision of integral services encompassing gender‐affirming care can facilitate receipt of other health services and are paramount to shaping health outcomes among transgender people [54, 55, 56, 57, 58, 59, 60]. Despite this evidence, few such models exist in India.

To address this implementation gap and to intervene on the inequities experienced in health services by transgender people, starting in February 2021, we established three comprehensive person‐centred clinics, known as “*Mitr*” clinics, in the Indian cities of Hyderabad (Telangana State), Pune and Thane (Maharashtra State). We detail the implementation of these clinics and provide evaluation insights on (1) the delivery and uptake of HIV and gender‐affirming services; (2) the association of receiving gender‐affirming services and other factors such as socio‐demographic characteristics and risk behaviours with HIV testing receipt; and (3) the HIV prevention and treatment cascades among clients.

## 2 METHODS

### 2.1 Implementation of *Mitr* clinics

*Mitr* clinics were established on foundational principles: (1) gender‐affirming person‐centred care as a fundamental right; (2) integration of medical, psychosocial and legal services to minimize barriers; (3) ownership of the clinic by members of the community in the cities where they were established.

Initial implementation processes included multiple rounds of consultations with community members and stakeholders in each city on service delivery priorities across the spectrum of person‐centred care, including health, psychosocial, legal and advocacy needs. A discussion guide was developed to elicit stakeholders’ perspectives. Key service priorities recommended included: (1) integration of HIV care, gender‐affirming services and support to access legal services and social protection schemes in decentralized community‐based, “one‐stop” venues; (2) expansion of counselling services beyond HIV; and (3) enhancement of peer navigation. Consensus regarding which of the service delivery priorities could be implemented across *Mitr* clinics was achieved through discussions between our team and community leaders. Clinics ensured that transgender personnel were represented at multiple staffing levels, offering a credible and comfortable community‐led environment while providing a pathway for professional development for staff. Each *Mitr* clinic includes a physician, clinic manager, nurse, a peer counsellor and two community outreach workers.

Gender‐affirming services at *Mitr* clinics include consultations and prescriptions for gender‐affirming hormone therapy (GAHT), laser hair removal services, mental health services (including individual and family counselling, substance use counselling, referrals to psychiatrists and other mental health professionals), counselling on surgical services (top surgery, breast implants and other gender‐affirming surgery), and social and legal gender identity services (i.e. assistance with transgender certificate applications and accessing state welfare benefits). HIV and sexual health services include rapid on‐site HIV screening with linkage to government integrated counselling and testing centres to receive confirmatory testing, provision of PrEP (which was initiated across *Mitr* clinics in October 2022), testing for syphilis (as per local guidelines) [61], and provision of lubricants and condoms. Syndromic management of sexually transmitted infections (STIs) is the standard in the public sector in India, with syphilis being the only STI with an on‐site rapid test [61]. While all the aforementioned services are provided on site either free of cost or at subsidized rates (i.e., laser hair removal services), ART and surgical services are facilitated through referrals to government ART centres and surgical programmes at government or private hospitals. Clients paid out‐of‐pocket for surgical services and GAHT.

### 2.2 Evaluation of uptake of services at *Mitr* clinics

#### 2.2.1 Data collection

Evaluation of *Mitr* clinics included both programmatic data routinely collected at all *Mitr* clinics as well as a one‐time semi‐structured survey collected at one site. Programmatic data were collected between 1 March 2021, and 30 September 2024 (*n* = 5226 clients registered during this time). All programmatic data were stored in electronic medical records. Variables pertaining to socio‐demographics, reported risk behaviours, services provided, HIV and syphilis testing history, PrEP delivery, ART and viral load testing were retained for analysis. Of note, during registration, clients were queried on substance use, and the Alcohol Use Disorders Identification Tool (AUDIT‐C) was administered. Semi‐structured interviews were conducted between 28 June 2022, and 28 July 2022, in Hyderabad as part of continuous quality improvement. In‐person interviews were conducted with 52 clients and 7 staff. The Hyderabad *Mitr* clinic was selected as it had been operational for over a year at the time. Clients invited to participate in the interviews were selected from those visiting Hyderabad's clinic in June–July 2022. To understand barriers and facilitators of HIV testing, clients were purposively invited by HIV testing uptake, with about half having received a test at the clinic. The interview guide included close‐ended and open‐ended questions and explored (1) reasons for interest in *Mitr* clinics and services broadly, and (2) barriers and facilitators of HIV testing. Interviews were conducted in a private room at the clinic and lasted 30 minutes. No compensation was provided.

#### 2.2.2 Definitions

*Eligibility for HIV testing*: Clients were deemed eligible to receive HIV testing if they had never received HIV testing, had previously tested negative or did not know their HIV status.

*Eligibility for PrEP*: Eligibility for PrEP was defined per national guidelines [62], namely a negative HIV rapid antibody test on the day of PrEP initiation, substantial risk for HIV acquisition by self‐report, no contraindication to PrEP, assessed as ready to initiate PrEP and willing to attend follow‐up evaluations.

*PrEP persistence*: PrEP persistence was defined as obtaining medication refills, without an interruption for > 30 days for ≥ 3 consecutive months, consistent with other literature [63].

*Retention in HIV care*: Retention in HIV care was defined as receipt of ART refills without interruptions and within 28 days of the expected refill date per national guidelines [64].

#### 2.2.3 Data analysis

We used summary statistics to describe socio‐demographic characteristics, service utilization, and HIV prevention and treatment continuum measures across all three *Mitr* clinics. To evaluate the association between receipt of gender‐affirming services, socio‐demographic characteristics and risk behaviours with HIV testing, we utilized multivariable logistic regression models among clients eligible for HIV testing. Covariates included socio‐demographic characteristics such as age, gender identity, marital status, education, prior history of HIV testing, behavioural factors (e.g. recent drug use, alcohol use, recent sexual intercourse) and receipt of one or more gender‐affirming services at *Mitr* clinics. Factors significantly associated with HIV testing in univariable analysis were considered for inclusion in multivariable analysis. Covariates that retained statistical significance (*p* < 0.05) were included in the final model. Multicollinearity was assessed using the variance inflation factor, and a clustered sandwich estimator was used to account for clustering within clinics. All quantitative analyses were conducted using Stata version 17 (StataCorp, 2021). To categorize open‐ended responses from semi‐structured interviews, we employed principles of text analysis [65] and manually collated responses using Microsoft Excel 2021 (Microsoft Corporation, 2021).

### 2.3 Ethical clearances

Interviewers administered informed oral consent in the local language of participants prior to asking questions for the semi‐structured interviews, explaining the voluntary nature of the interviews. No identifying information was collected during the interviews. The use of programmatic data at the *Mitr* clinics is approved by the Johns Hopkins University IRB as public health surveillance.

## 3 RESULTS

### 3.1 Socio‐demographic characteristics and self‐reported risk behaviours among clients

Among the 5226 unique clients who registered between 1 March 2021, and 30 September 2024 (Table 1), the median age of clients was 26 years (interquartile range [IQR] 22–30 years). Ninety‐six percent of clients were assigned male sex at birth. The majority of clients (86%) self‐identified as TGW, while 5% as transgender men (TGM), and 7% as “other gender.” Most clients (71%) had high school or lower education and were unmarried (89%). A quarter (25%) reported earning wages through begging, while 21% earned wages through performing “badhai” (a cultural practice of providing blessings at weddings or when a child is born), and 20% reported being unemployed. Although only 16% of clients identified sex work as their current occupation, over a third (35%) reported a history of transactional sex.

**Table 1:** Client demographics and sexual/substance use behaviours at registration among 5226 clients receiving services at *Mitr* clinics across three cities in India from 1 March 2021 to 30 September 2024

Clients registered	Total		Hyderabad		Pune		Thane	
***N***	5226		2082		1511		1633	
Age, years								
Median (IQR)	26 (22–30)		25 (22–30)		26 (23–30)		26 (22–31)	
Gender^a^	*n*	%	*n*	%	*n*	%	*n*	%
Man	126	2	74	4	41	3	11	1
Woman	7	0.1	6	0.3	0		0	
TGM	239	5	88	4	86	6	65	4
TGW	4472	86	1889	91	1114	74	1472	90
Other	377	7	23	1	269	18	85	5
Unknown	2	0.04	2	0.1	0		0	
Sex‐assigned at birth								
Male	5001	96	1985	95	1430	95	1586	97
Female	217	4	92	4	78	5	47	3
Intersex	7	0.1	5	0.2	2	0.1	0	
Refused	1	0.02	0		1	0.1	0	
Registered at a TI programme								
No	3641	70	1363	65	1117	74	1161	71
Yes	872	17	215	10	191	13	466	29
Unknown	713	14	504	24	203	13	6	0.4
Primary occupation								
Unemployed	1055	20	299	14	425	28	331	20
Employed	276	5	66	3	114	8	96	6
Sex work	829	16	398	19	329	22	102	6
Badhai	1086	21	711	34	96	6	279	17
Begging	1332	25	301	14	383	25	648	40
Dancing	79	2	24	1	17	1	38	2
Other	373	7	206	10	96	6	71	4
Unknown	196	4	77	4	51	3	68	4
Education level								
No school	812	16	310	15	257	17	245	15
Primary education	463	9	172	8	85	6	206	13
Middle education	703	13	230	11	134	9	339	21
Higher secondary	1745	33	695	33	515	34	535	33
Graduate	1334	26	585	28	489	32	280	17
Other	15	0.3	4	0.2	7	0.5	4	0.2
Unknown	154	3	86	4	44	3	24	1
Marital status								
Unmarried	4629	89	1708	82	1366	90	1555	95
Married	367	7	262	13	70	5	35	2
Divorced/separated/widowed	63	1	32	1.5	6	0.4	25	2
Live in partner	3	0.1	1	0.05	1	0.1	1	0.1
Unknown	164	3	79	4	68	5	17	1
Sexually active in the past 12 months								
No	1097	21	371	18	98	6	628	38
Yes	3999	77	1658	80	1361	90	980	60
Unknown	130	2	53	3	52	3	25	2
Number of sexual partners in the past 12 months (among those sexually active, *n* = 3999)								
Median (IQR)	3 (1–15)		4 (1–100)		2 (1–5)		4 (1–13)
Engaged in sex work currently								
No	2236	56	893	54	695	51	648	66
Yes	1385	35	731	44	426	31	228	23
Unknown	378	9	34	2	240	18	104	11
Years in sex work (*n* = 1385)								
Median (IQR)	3 (2–6)		4 (2–8)		3 (2–5)		2 (1–5)
Sex acts per day in the last week (among those in sex work, *n* = 1385)								
Median (IQR)	5 (2–10)		4 (2–10)		5 (3–10)		7 (4–12)
Current alcohol use								
No	2872	55	966	46	787	52	1119	69
Yes	2077	40	1043	50	655	43	379	23
Unknown	277	5	73	4	69	5	135	8
AUDIT‐C Score (*n* = 2077)								
Moderate/low risk	1772	85	998	96	517	79	257	68
Severe/high risk	305	15	45	4	138	21	122	32
Injection drug use								
Never	4637	89	1974	95	1238	82	1435	88
In the last 6 months	12	0.2	5	0.2	3	0.2	4	0.2
Over 6 months ago	6	0.1	2	0.1	3	0.2	1	0.1
Unknown	561	11	101	5	267	18	193	12
Non‐injection drug use in the last 6 months								
No	3399	65	1861	89	564	37	974	60
Yes	117	2	102	5	15	1	0	
Unknown	1710	33	119	6	932	62	659	40
On GAHT								
Yes, currently	189	4	74	4	19	1	96	6
No, but have in the past	351	7	169	8	73	5	109	7
No	3995	76	1620	78	993	66	1382	85
Unknown	691	13	219	11	426	28	46	3
Ever shared needles for GAHT (*n* = 540)								
No	169	31	145	60	11	12	13	6
Yes	6	1	3	1	1	1	2	1
Unknown	365	68	95	39	80	87	190	93
HIV status at registration								
Positive	331	6	202	10	100	7	29	2
Tested in the last 6 months	2222	43	888	43	683	45	651	40
Tested over 6 months ago	849	16	390	19	287	19	172	11
Never tested	1401	27	496	24	239	16	666	41
Unknown	423	8	106	5	202	13	115	7

Abbreviations: AUDIT‐C, Alcohol Use Disorders Identification Test; GAHT, gender‐affirming hormone therapy; TGM, transgender men; TGW, transgender women; TI, Targeted Intervention.

^a^ Gender is reported as self‐identified by the client.

Three‐quarters of clients (77%) reported recent sexual intercourse in the prior 12 months. Among those who reported recent sexual intercourse, the median number of sexual partners was 3 (IQR: 1–15 partners). Among those who reported a history of transactional sex in the prior 12 months, the median number of sex partners was 24 (IQR: 3–350). Over a third of clients (40%) reported alcohol use, with 15% being classified at high or severe risk for alcohol use disorders based on the AUDIT‐C [66]. Only 2% of clients reported any history of substance use other than alcohol. City‐specific data on socio‐demographic characteristics and self‐reported behaviours are presented in Table 1.

### 3.2 Service utilization at *Mitr* clinics

Seventy percent of clients who registered at *Mitr* clinics had never been registered in the public sector TI programme by self‐report. At registration, only 10% of clients were currently on or reported prior receipt of GAHT. Most clients (75%) received gender‐affirming care services, with the majority (53%) accessing laser hair removal services (Table 2). While 34% received consultations and prescriptions for GAHT, 26% received referrals to surgical services, and 15% received social and legal gender identity services. Most clients who received counselling services primarily received HIV pre‐ and post‐test and risk reduction counselling (78%). Additionally, 8% of clients also received individual mental health, substance use and family counselling services. Clients also accessed other HIV prevention services, with 38% receiving condoms.

**Table 2:** Utilization of services at *Mitr* clinics across three Indian cities from 1 March 2021 to 30 September 2024 (*N* = 5226)

	Total		Hyderabad		Pune		Thane	
Services	*n*	%	*n*	%	*n*	%	*n*	%
Screened for syphilis	1059	20	755	36	158	10	146	9
Risk reduction counselling services	1413	27	699	34	562	37	152	9
HIV testing and/or HIV counselling services	4071	78	1580	76	1308	87	1183	72
HIV motivational counselling	2387	46	467	22	848	56	1072	66
Mental health services	375	7	295	14	30	2	50	3
Gender‐affirming hormone therapy services	1790	34	727	35	784	52	279	17
Family counselling services	156	3	152	7	0		4	0.2
Gender‐affirming surgery services	943	18	293	14	401	27	249	15
Breast implant services	735	14	133	6	388	26	214	13
Reproductive health services	22	0.4	5	0.2	17	1	0	
Sexual health services	48	1	1	0.5	44	3	3	0.2
Laser hair removal services	2794	53	893	43	647	43	1254	77
Gender identity services (i.e. transgender identity cards)	758	15	198	10	373	25	187	11
Condoms	1996	38	894	43	138	9	964	59
Lube	232	4	231	11	0		1	0.1
General PrEP counselling	1332	25	520	25	191	13	621	38
PEP counselling	65	1	58	3	2	0.3	5	0.3
Top surgery counselling	71	1	14	1	31	2	26	2
Substance use counselling	186	4	160	8	26	2	0	
Other services	1946	37	1093	52	495	33	358	22

Abbreviations: PEP, post‐exposure prophylaxis; PrEP, pre‐exposure prophylaxis.

### 3.3 HIV testing cascade and correlates of HIV testing

**Figure 1:**
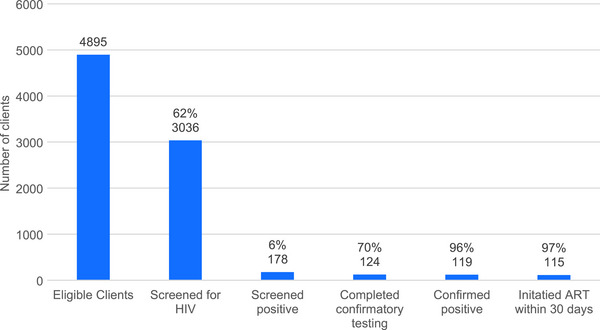
HIV testing cascade at the three *Mitr* clinics across India (Hyderabad, Pune and Thane) between 1 March 2021 and 30 September 2024. *Note*: Denominator for percentages are the number satisfying prior indicator; for example 3036 (62%) out of the 4895 eligible clients were screened for HIV. Abbreviation: ART, antiretroviral therapy.

Among the 5226 clients who registered at *Mitr* clinics, 4895 were eligible to receive HIV testing. Among eligible clients, 3036 (62%) were screened for HIV using rapid test kits, of whom 178 (6%) had screened positive (Figure 1). Among the 178 clients who had a positive HIV screening test, 124 (70%) completed confirmatory testing at government integrated counselling and testing centres, of whom 119 (96%) were confirmed as being HIV positive (Figure 1). Thus, 3.92% of all eligible clients screened for HIV were confirmed as being HIV positive.

In multivariable analysis, recent sexual activity (aOR 1.60, 95% CI 1.06–2.40), high/severe risk for alcohol use disorders (aOR 1.37, 95% CI 1.22–1.53), having a long‐term partner (aOR 2.96, 95% CI 1.22–7.22) and seeking referrals for surgical services (aOR 1.51, 95% CI 1.02–2.25) were significantly associated with greater odds of HIV testing. Conversely, age > 25 years, gender self‐identified as man, low/moderate risk of alcohol use disorders and being married were significantly associated with lower odds of HIV testing (all *p* < 0.05; Table 3). No multicollinearity was identified among variables included in the multivariable model.

**Table 3:** Correlates of receiving an HIV test at the three *Mitr* clinics across India (*N* = 4786)

	Univariable			Multivariable		
HIV screening test	Coef.	[95% Conf. Interval]		Coef.	[95% Conf. Interval]	
**Gender**						
Man	0.67^**^	0.50	0.90	0.55^**^	0.39	0.78
Woman	0.26	0.05	1.29	0.17	0.03	1.18
TGM	1.09	0.47	2.53	0.74	0.51	1.08
TGW	*ref*.					
Other/unknown	1.90^**^	1.30	2.78	1.51^**^	1.12	2.05
**Sex**						
Male	*ref*.					
Female	0.90	0.43	1.96			
Intersex	0.95	0.45	2.61			
**Age group**						
25 or less	*ref*.					
26 or older	0.69^***^	0.61	0.79	0.73^***^	0.65	0.81
Unknown	0.85	0.71	1.02	1.43	0.97	2.10
**Job**						
Unemployed	*ref*.					
Employed	1.25^***^	1.14	1.37	1.57^***^	1.35	1.82
Sex work	0.95	0.47	1.91	1.09	0.69	1.70
Badhai	0.54	0.29	1.01	0.72	0.50	1.04
Begging	0.79	0.47	1.32	0.91	0.60	1.37
Dancing	1.00	0.71	1.40	1.13	0.88	1.45
Other	0.60^**^	0.44	0.81	0.74	0.53	1.03
**Education level**						
Secondary school or below	*ref*.					
Above secondary schooling	1.29^**^	1.07	1.54			
Other	0.62	0.32	1.21			
**Marital status**						
Unmarried	*ref*.					
Married	0.52^***^	0.44	0.61	0.60	0.50^***^	0.71
Divorced/separated/widow	0.68^***^	0.65	0.72	0.99	0.91	1.08
Live in partner	2.90^***^	1.85	4.56	2.96	1.22^*^	7.22
Unknown/refused	0.48^*^	0.26	0.90	0.74	0.21	2.59
**HIV testing history at registration**						
Tested negative in the last 6 months	*ref*.					
Tested negative over 6 months ago	1.49^**^	1.14	1.96	1.46^***^	1.17	1.83
Never tested	2.52	0.66	9.62	2.54	0.80	8.11
Unknown/refused	0.91	0.29	2.87	1.08	0.30	3.90
**Sexually active**						
No	*ref*.					
Yes	1.62^**^	1.23	2.13	1.60^*^	1.06	2.40
Unknown	0.65^*^	0.42	0.99	0.62^***^	0.51	0.77
**Sex work**						
No	*ref*.					
Yes	0.97	0.59	1.59			
**AUDIT‐C rating**						
No alcohol use	*ref*.					
Low/moderate risk	0.86^*^	0.76	0.97	0.84^***^	0.76	0.92
Severe/high risk	1.44^**^	1.10	1.87	1.37^***^	1.22	1.53
Unknown	0.96	0.74	1.26	1.62^**^	1.14	2.29
**Any drug use**						
No	*ref*.					
Yes	1.00	0.55	1.83			
**Gender‐affirming surgery services** ^a^						
No	*ref*.					
Yes	1.56^*^	1.02	2.40	1.51^*^	1.02	2.25
**Gender identity services (i.e. transgender identity cards)**						
No	*ref*.					
Yes	0.98	0.83	1.16			
**Gender‐affirming hormone therapy**						
No	*ref*.					
Yes	1.27	0.69	2.35			
**Laser hair removal services**						
No	*ref*.					
Yes	0.99	0.96	1.02			

Abbreviations: AUDIT‐C, Alcohol Use Disorders Identification Test; TGM, transgender men; TGW, transgender women.

^a^ Gender‐affirming surgery services include services related to gender‐affirming surgery, top surgery and breast implants.

*** indicates a *p*‐value < 0.001.

** indicates a *p*‐value < 0.01.

* indicates a *p*‐value < 0.05.

### 3.4 HIV prevention and treatment cascades

**Figure 2:**
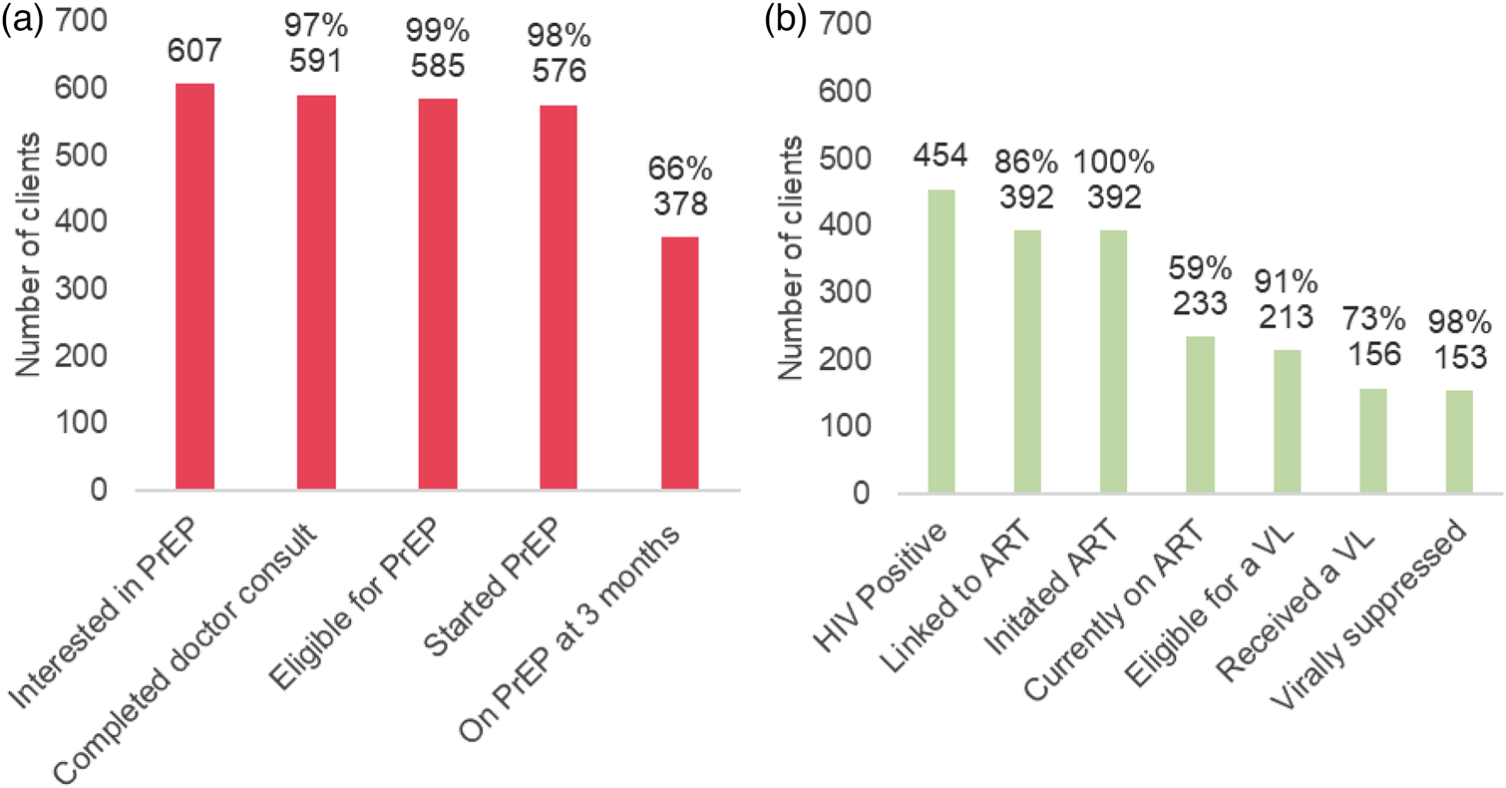
Pre‐exposure prophylaxis (PrEP) (Panel A) and HIV care cascade (Panel B) among clients at the three *Mitr* clinics across India from 1 March 2021 to 30 September 2024. *Note*: Denominator for percentages are the number satisfying prior indicator; for example 591 (97%) out of the 607 clients interested in PrEP completed a doctor consultation. Abbreviations: ART, antiretroviral therapy; PrEP, pre‐exposure prophylaxis; VL, viral load.

Among 607 *Mitr* clinic clients interested in PrEP, 591 (97%) completed a consultation with the physician; 585 (99%) were eligible per national guidelines [62]. Of these, 576 clients (98%) initiated PrEP, of whom 378 (66%) were PrEP persistent (Figure 2). One seroconversion was observed among clients initiating PrEP. The most common reasons for PrEP discontinuation reported were perception of being at low risk (51%) or being no longer interested (19%).

For ART initiation and viral suppression, we report available data from clients *newly* diagnosed with HIV through initial screening at *Mitr* clinics, as well as clients known to be living with HIV at registration. Among 119 clients newly diagnosed with HIV after registration, 115 (97%) initiated ART within 30 days (Figure 1). Among 454 HIV clients with HIV (including those previously diagnosed with HIV), 392 (86%) had initiated ART. As of 30 September 2024, 233 of these clients (59%) were still engaged in *Mitr* clinic services and retained in HIV care; data on HIV care engagement for the remainder of the clients were not systematically available at the *Mitr* clinics. Among those ascertained to still be engaged in HIV care, 213 were eligible for a viral load (i.e. those who initiated ART over 6 months ago), of whom 156 (73%) had ever received a viral load test, and 153 (98%) were virally suppressed (viral load <1000 copies/ml).

3.5 | Perspectives from clients and providers at Hyderbad's *Mitr* clinic Among 52 clients who completed the semi‐structured survey in Hyderabad's *Mitr* clinic, most (83%) had first heard about the clinic from their friends and/or clinic providers themselves, while 17% had learned about the clinic from social media. Accessibility to a variety of gender‐affirming services and receiving services from providers from the community were cited as the main reasons for interest in the clinic.

Specific to HIV testing, clinic providers reported conducive workflows with opportunities to offer HIV testing to clients at registration as well as when clients presented to receive other non‐HIV services. Providers and clients identified trust fostered by service provision by members of the community as a key facilitator of HIV testing uptake. At the same time, both groups identified barriers encountered by the subset of clients who declined HIV testing despite being eligible. Providers perceived lack of knowledge, HIV stigma and fear of HIV status disclosure, especially to older members of the community (also known as “gurus”) as the main reasons for declining HIV testing. Half of the eligible clients who declined HIV testing perceived themselves at low risk for HIV acquisition.

With regard to the HIV testing cascade and linkage to government ART centres, clients identified fragmented services as a main barrier. Specifically, clients reported the need to receive confirmatory HIV testing at a different venue (i.e. government integrated testing and counselling centres) after initial HIV screening at *Mitr* clinics, and unfriendly staff at those centres as hampering their enthusiasm. Providers additionally reported challenges in obtaining reliable contact information from clients with HIV to facilitate linkage to government ART centres due to clients’ disclosure fears.

## 4 DISCUSSION

The *Mitr* clinics represent one of the first community‐based, person‐centred service delivery models integrating gender‐affirming services with HIV services for transgender people in India. Key findings include the notable role that gender‐affirming services delivered by transgender providers played in providing an entry portal for HIV services among clients. We also observed high uptake of PrEP among interested clients and retention in HIV care among clients with HIV. One of the key challenges observed was the fragmented service delivery with respect to ART and confirmatory testing, which, if addressed, could further improve HIV outcomes.

The low engagement of transgender people in HIV services is consistent with other Indian studies [38, 67, 68, 69]. In a recent nationwide survey among sexually active transgender and gender diverse individuals, nearly 50% had never accessed HIV testing [67]. The *Mitr* clinics highlight that co‐locating gender‐affirming services can significantly enhance this reach. Most notably, the majority of clients who registered at *Mitr* clinics had never engaged with TI programmes; about 75% of clients who accessed *Mitr* clinics did so to receive gender‐affirming care. Co‐locating gender‐affirming care with HIV services not only promoted initial engagement but also presented multiple contact points to offer HIV testing and PrEP. Additionally, staffing from the community was equally integral. These factors likely led to a majority of eligible clients receiving an HIV test, and almost all clients interested in PrEP initiating PrEP. In fact, receipt of gender‐affirming services was associated with a higher likelihood of receiving an HIV test. We did not observe a similar uptake with STI testing, suggesting a potential use case for HIV/syphilis dual kits. Collectively, these findings suggest that person‐centred care models can be compelling entry points for the receipt of HIV services among transgender people in India.

Findings from *Mitr* clinics are also consistent with findings from the evaluation of care models in other low‐ and middle‐income countries (LMICs) delivering HIV services to transgender people. In South Africa, receipt of GAHT was associated with viral suppression [70]. In Vietnam, integrating gender‐affirming services delivered by transgender providers increased PrEP uptake and persistence [71]. In Thailand, “The Integrated Trans Model” clinics, providing primary care, HIV and gender‐affirming services, have demonstrated low HIV incidence [72]. Dissemination of The Integrated Trans Model to other Asian countries has led to increased HIV and PrEP services access among TGW [73]. Contrastingly, a randomized trial in the United States and Brazil [74] found high rates of PrEP acceptance and persistence regardless of the co‐location of gender‐affirming services. However, participants in the trial were referred to pre‐identified centres with well‐established gender‐affirming services, and had access to experienced peer navigators. Unlike these sites, in the *Mitr* clinic cities, there were no other alternative venues for the receipt of comprehensive gender‐affirming care. Our findings emphasize that in settings with with limited access to gender‐affirming services, co‐locating gender‐affirming services with HIV services and reducing barriers to integrated care is paramount to engaging transgender people.

While *Mitr* clinics have been successful in achieving initial engagement and uptake of HIV testing and PrEP, we observed some attrition. Clients had to present to separate venues to receive confirmatory HIV testing and ART, respectively. Although these venues were in proximity to *Mitr* clinics, and outreach workers navigated clients to these venues, 39% of clients with a positive HIV screening test did not complete confirmatory testing. This confirmatory test at a public sector facility is required to access public sector ART. Psychosocial factors such as HIV stigma and fear of HIV status disclosure were the main reasons for declining HIV testing, while low perceived HIV risk was a prominent reason for declining HIV testing as well as discontinuing PrEP. HIV status disclosure concerns, in addition to fragmented HIV treatment services, also made it challenging to track ongoing ART receipt and viral load measurements (performed yearly at government ART centres) among clients with HIV. Therefore, our estimates of the proportion of clients with HIV continuing to receive ART need to be interpreted with caution—it is likely that a larger proportion are currently on ART as they may be seeking care without accessing *Mitr* clinics’ resources. Viral suppression was high among those ascertained to be taking ART and had received viral load measurements. Decentralizing public sector ART initiation and confirmatory testing to community‐based settings, such as *Mitr* clinics, with a large volume of people with HIV, could help improve HIV testing and treatment outcomes. Other non‐clinic‐based strategies we have evaluated, such as confidential virtual platforms coupled with individualized support from virtual counsellors, have also demonstrated improved HIV testing among gender and sexual minority populations in India [75].

Finally, in the context of the 2019 Transgender Persons (Protection of Rights) Act, there is considerable interest in the development of comprehensive care models for transgender people. The National AIDS Control Organization has detailed the delivery of comprehensive services to transgender people as a key priority in a recently published forward‐facing white paper [76]. The *Mitr* clinics are highlighted as an illustrative example and represent among the only community‐based single window venues to receive both gender‐affirming services and HIV services [76]. Drawing from our insights from *Mitr* clinics, co‐locating gender‐affirming services with TI programmes, integrated counselling and testing centres, and government ART centres, and building a workforce that includes transgender staff in these venues could offer a sustainable pathway to engage transgender people in public sector HIV services. At the same time, scale‐up of community‐based models like *Mitr* clinics could also be effective in improving population‐level HIV prevention and treatment outcomes among transgender people in India. Most notably, this scale‐up has already occurred with government investment in the state of Telangana.

The findings from our evaluation of *Mitr* clinics are not without limitations. While the principles of person‐centred care would apply broadly, our findings may not be generalizable to all transgender people in India. For example, we observed low injection drug use among clients in the *Mitr* clinics; this finding may not be generalizable to cities in Northeast India, where injection drug use in general is more prevalent. Surveys on client and provider perspectives were only administered at a single clinic site, further limiting generalizability. Also, of importance, our analyses were limited to data routinely collected for programmatic and clinical needs, which resulted in missing data, limiting our ability to fully characterize variables such as HIV care retention and viral load testing on all clients with HIV, PrEP discontinuation reasons and utilization of psychosocial services. Our findings also have several strengths, including the systematic collection of programmatic data over 3 years and the inclusion of data from multiple cities.

## 5 CONCLUSIONS

As a single window model of community‐based, person‐centred care delivery to transgender people, *Mitr* clinics have greatly increased accessibility of gender‐affirming services. The clinics successfully reached previously unengaged transgender people. Expansion of this model with the integration of confirmatory HIV testing and ART on site can ameliorate the disparities that transgender people experience in receipt of both gender‐affirming services and HIV services, and holds promise for shaping the HIV epidemic in India.

## COMPETING INTERESTS

SSS serves as the Managing Trustee of the YR Gaitonde Medical Educational and Research Foundation, which is the entity that oversees YRGCARE. SSS serves on the Board of Directors of the Serious Fun Children's Network. SSS has also received grants/products to the Institution from Abbott Laboratories and Gilead Sciences, not related to this manuscript. KHM receives grants to the institution from Viiv, Gilead and Merck, not related to this manuscript, and serves on the Scientific Advisory Board for Viiv, Gilead Sciences and Merck that are not related to this manuscript. All other authors have nothing to disclose.

## AUTHORS' CONTRIBUTIONS

SS, AS, KHM, SHM and SSS conceived the concept of the *Mitr* clinic. SS, PMR, KP, AE, VA, SO, AS and SSS led the implementation of the clinics with support from SKP and SK. JB, AE, VA, AMM, SHM, RPK and SO developed the database and survey instruments. AK, KA, KHM and SSS provided technical assistance to the delivery of services at the *Mitr* clinics. SS, KHM, SSS and LG conceived the manuscript. LG drafted the manuscript with inputs from SSS, AMM and SHM. All authors reviewed the manuscript and provided critical feedback.

## FUNDING

The *Mitr* clinics are funded by PEPFR/USAID India Cooperative Agreement Number: 72038619CA00001.

## Data Availability

The data that support the findings of this study are available on request from the corresponding author. The data are not publicly available due to privacy or ethical restrictions.
